# Glycine–d-tartaric acid (1/1)

**DOI:** 10.1107/S1600536813000822

**Published:** 2013-01-16

**Authors:** T. Mohandas, C. Ranjith Dev Inbaseelan, S. Saravanan, P. Sakthivel

**Affiliations:** a Department of Physics, Shri Angalamman College of Engineering and Technology, Siruganoor, Tiruchirappalli 621 105, India; b Centre for Photonics and Nanotechnology, Sona College of Technology, Salem, Tamilnadu, India; c Department of Physics, Urumu Dhanalakshmi College, Tiruchirappalli 620 019, India

## Abstract

In the title co-crystal, C_2_H_5_NO_2_·C_4_H_6_O_6_, the gylcine mol­ecule is present in the zwitterion form. In the tartaric acid mol­ecule there is a short intra­molecular O—H⋯O contact. In the crystal, the tartaric acid mol­ecules are linked *via* pairs of O—H⋯O hydrogen bonds, forming inversion dimers. These dimers are linked *via* a number of O—H⋯O and N—H⋯O hydrogen bonds involving the two components, forming a three-dimensional network.

## Related literature

For related structures, see: Kvick *et al.* (1980 ▶). For a description of the Cambridge Structural Database, see: Allen (2002 ▶).
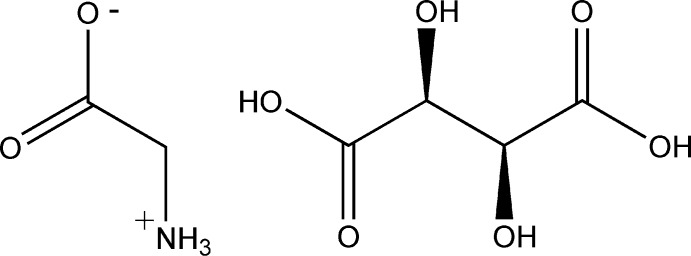


## Experimental

### 

#### Crystal data

C_2_H_5_NO_2_·C_4_H_6_O_6_
*M* *_r_* = 225.16
Monoclinic, 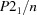

*a* = 4.8387 (2) Å
*b* = 9.2913 (4) Å
*c* = 20.0273 (8) Å
β = 90.171 (1)°
*V* = 900.38 (6) Å^3^
*Z* = 4
Mo *K*α radiation
μ = 0.16 mm^−1^
*T* = 293 K
0.30 × 0.20 × 0.20 mm

#### Data collection

Bruker Kappa APEXII diffractometer
Absorption correction: multi-scan (*SADABS*; Bruker, 2003 ▶) *T*_min_ = 0.954, *T*_max_ = 0.969
12500 measured reflections
3282 independent reflections
2685 reflections with *I* > 2σ(*I*)
*R*_int_ = 0.033

#### Refinement

*R*[*F* ^2^ > 2σ(*F* ^2^)] = 0.037
*wR*(*F* ^2^) = 0.112
*S* = 1.07
3282 reflections
165 parameters
H atoms treated by a mixture of independent and constrained refinement
Δρ_max_ = 0.49 e Å^−3^
Δρ_min_ = −0.22 e Å^−3^

### 

Data collection: *APEX2* (Bruker, 2004 ▶); cell refinement: *APEX2* and *SAINT-NT* (Bruker, 2004 ▶); data reduction: *SAINT-NT* and *XPREP* (Bruker, 2003 ▶); program(s) used to solve structure: *SHELXS97* (Sheldrick, 2008 ▶); program(s) used to refine structure: *SHELXL97* (Sheldrick, 2008 ▶); molecular graphics: *ORTEP-32* (Farrugia, 2012 ▶); software used to prepare material for publication: *PLATON* (Spek, 2009 ▶).

## Supplementary Material

Crystal structure: contains datablock(s) global, I. DOI: 10.1107/S1600536813000822/bv2215sup1.cif

Structure factors: contains datablock(s) I. DOI: 10.1107/S1600536813000822/bv2215Isup2.hkl

Additional supplementary materials:  crystallographic information; 3D view; checkCIF report

## Figures and Tables

**Table 1:** Hydrogen-bond geometry (Å, °)

*D*—H⋯*A*	*D*—H	H⋯*A*	*D*⋯*A*	*D*—H⋯*A*
N1—H1*A*⋯O1^i^	0.953 (19)	2.20 (2)	2.9509 (11)	135.2 (16)
N1—H1*A*⋯O3^i^	0.953 (19)	2.21 (2)	2.9386 (11)	132.2 (15)
N1—H1*B*⋯O6^ii^	0.909 (16)	2.041 (16)	2.9188 (10)	162.0 (14)
N1—H1*C*⋯O7^ii^	0.914 (18)	2.172 (17)	2.9492 (13)	142.4 (14)
O2—H2*A*⋯O8^iii^	0.93 (2)	1.64 (2)	2.5473 (8)	167 (2)
O3—H3*A*⋯O4^iv^	0.870 (18)	1.849 (18)	2.7122 (8)	171.0 (16)
O4—H4*A*⋯O3^v^	0.81 (2)	2.09 (2)	2.7654 (10)	141.1 (18)
O4—H4*A*⋯O6	0.81 (2)	2.251 (18)	2.6743 (9)	112.9 (16)
O5—H5⋯O7	0.944 (19)	1.612 (19)	2.5459 (9)	169.2 (18)

Symmetry codes: (i) 
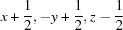
; (ii) 
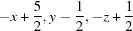
; (iii) 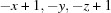; (iv) 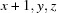; (v) 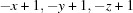.

**Fig. 1.**
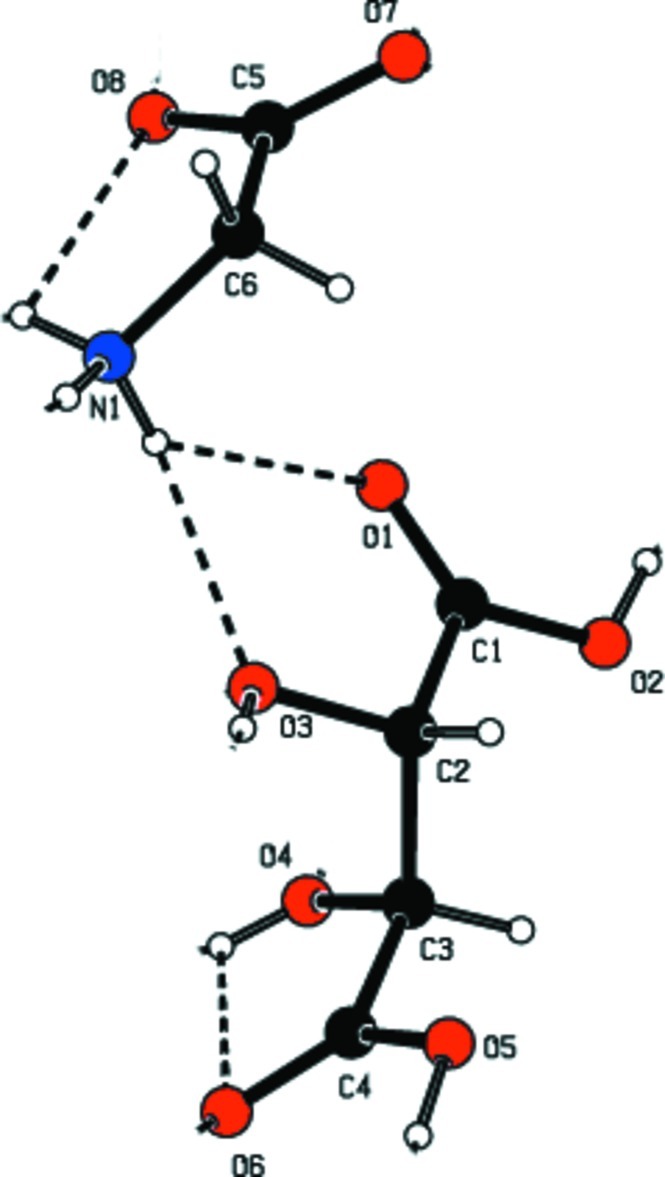
The molecular structure and labelling scheme for (I) with displacement ellipsoid of non-H atoms are drawn at the 30% probability level.

**Fig. 2.**
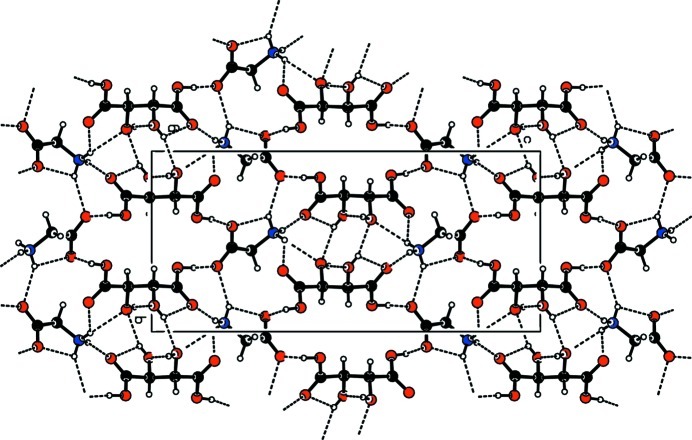
A packing diagram for (I) is shown. Dashed line indicates intra and inter molecular N—H..O and O—H..O hydrogen bonding interactions

## supplementary crystallographic information

### Comment

Glycine is the simplest aminoacid that is not optically active. It is essential for biosynthesis of nucleic acids as well as the biosynthesis of bile acids, creatine phosphate and other amino acids. Its geometric features of non covalent interactions at atomic resolution are important in the structural assembly and functions of proteins.

In the title compound(I), glycine is in the zwitterionic form. The tartaric acid molecule is in the un-ionized state. The angle between the planes of the half molecules O1/O2/C1/C2/O3 and O5/O6/C4/C3/O4 is 62.74 (3)°, which is closer to the value of 54.6° found in the structure of tartaric acid.

Atoms C5,C6,O7,N1 are planar with the N1 atom is slightly displaced out of this plane by -0.518 (1)°.

The relevant torsion angles are O7—C5—C6—N1 of -158.33 (3)° and O8—C5—C6—N1 of 23.08 (3)°. These can be compared with the corresponding values in pure Γ glycine 167.1 (1)° and -15.4 (1)°, respectively (Kvick *et al.*, (1980), which is more distorted from planarity.

The molecular structure of (I) is shown in the (Fig.1) and selected geometric parameters listed in Table 1. The bond lengths for C=N, *C*=O, C—C are within normal ranges (Allen 2002). The dihedral angle between planes of D-tartaric acid and glycine is 51.14 (9)°. The molecules related by the 2_1_ screw along *b* axis are linked by intermolecular O—H···O hydrogen bond generating a supramolecular chain.

The carbon skeleton of tartaric molecule is non-planar with a C1—C2—C3—C4 torsion angle of 177.8 (1)°. Fig.2 shows the packing diagram in which there are a large number of N—H···O and O—H···O hydrogen bonds.

### Experimental

Colourless single crystals were grown as transparent needles by slow evaporation method from a saturated aqueous solution containing glycine and D-tartaric acid in a 1:1 stoichiometric ratio.

### Refinement

All the hydrogen atoms were geometrically fixed and allowed to ride on their parent atoms with C—H = 0.97and 0.98 Å, and *U*_iso_ = 1.2_eq_(C). Hydrogen atoms attached to O and N were refined isotropically.

### Crystal data

**Table:**

C_2_H_5_NO_2_·C_4_H_6_O_6_	*F*(000) = 472
*M**_r_* = 225.16	*D*_x_ = 1.661 Mg m^−^^3^
Monoclinic, *P*2_1_/*n*	Mo *K*α radiation, λ = 0.71073 Å
Hall symbol: -P 2yn	Cell parameters from 1585 reflections
*a* = 4.8387 (2) Å	θ = 2.0–25.0°
*b* = 9.2913 (4) Å	µ = 0.16 mm^−^^1^
*c* = 20.0273 (8) Å	*T* = 293 K
β = 90.171 (1)°	Prism, colorless
*V* = 900.38 (6) Å^3^	0.30 × 0.20 × 0.20 mm
*Z* = 4	

### Data collection

**Table:**

Bruker Kappa APEXII diffractometer	2685 reflections with *I* > 2σ(*I*)
Radiation source: fine-focus sealed tube	*R*_int_ = 0.033
Graphite monochromator	θ_max_ = 35.0°, θ_min_ = 2.0°
ω and φ scan	*h* = −7→7
Absorption correction: multi-scan (*SADABS*; Bruker, 2003)	*k* = −14→12
*T*_min_ = 0.954, *T*_max_ = 0.969	*l* = −27→29
12500 measured reflections	2 standard reflections every 100 reflections
3282 independent reflections	intensity decay: none

### Refinement

**Table:**

Refinement on *F*^2^	Secondary atom site location: difference Fourier map
Least-squares matrix: full	Hydrogen site location: inferred from neighbouring sites
*R*[*F*^2^ > 2σ(*F*^2^)] = 0.037	H atoms treated by a mixture of independent and constrained refinement
*wR*(*F*^2^) = 0.112	*w* = 1/[σ^2^(*F*_o_^2^) + (0.0603*P*)^2^ + 0.1264*P*] where *P* = (*F*_o_^2^ + 2*F*_c_^2^)/3
*S* = 1.07	(Δ/σ)_max_ = 0.001
3282 reflections	Δρ_max_ = 0.49 e Å^−^^3^
165 parameters	Δρ_min_ = −0.22 e Å^−^^3^
0 restraints	Extinction correction: *SHELXL97* (Sheldrick, 2008), Fc^*^=kFc[1+0.001xFc^2^λ^3^/sin(2θ)]^-1/4^
Primary atom site location: structure-invariant direct methods	Extinction coefficient: 0.074 (5)

### Special details

**Table:**

Geometry. All esds (except the esd in the dihedral angle between two l.s. planes) are estimated using the full covariance matrix. The cell esds are taken into account individually in the estimation of esds in distances, angles and torsion angles; correlations between esds in cell parameters are only used when they are defined by crystal symmetry. An approximate (isotropic) treatment of cell esds is used for estimating esds involving l.s. planes.
Refinement. Refinement of F^2^ against ALL reflections. The weighted R-factor wR and goodness of fit S are based on F^2^, conventional R-factors R are based on F, with F set to zero for negative F^2^. The threshold expression of F^2^ > 2sigma(F^2^) is used only for calculating R-factors(gt) etc. and is not relevant to the choice of reflections for refinement. R-factors based on F^2^ are statistically about twice as large as those based on F, and R- factors based on ALL data will be even larger.

### Fractional atomic coordinates and isotropic or equivalent isotropic displacement parameters (Å^2^)

**Table:**

	*x*	*y*	*z*	*U*_iso_*/*U*_eq_	
C1	0.44851 (16)	0.24734 (9)	0.61943 (4)	0.02360 (17)	
C2	0.64567 (15)	0.25044 (9)	0.56018 (4)	0.02271 (17)	
H2	0.7688	0.1668	0.5624	0.027*	
C3	0.47689 (16)	0.24434 (9)	0.49514 (4)	0.02364 (17)	
H3	0.3788	0.1521	0.4936	0.028*	
C4	0.66706 (17)	0.25298 (9)	0.43493 (4)	0.02490 (18)	
C5	1.19172 (16)	0.03299 (10)	0.29340 (4)	0.02399 (18)	
C6	1.42473 (16)	0.05011 (10)	0.24364 (4)	0.02740 (19)	
H6A	1.4296	0.1487	0.2278	0.033*	
H6B	1.5996	0.0297	0.2655	0.033*	
N1	1.38776 (19)	−0.04805 (10)	0.18645 (4)	0.03233 (19)	
O1	0.44118 (15)	0.33804 (9)	0.66214 (4)	0.03694 (19)	
O2	0.29078 (17)	0.13348 (8)	0.61631 (4)	0.0395 (2)	
O3	0.80456 (13)	0.37788 (7)	0.56436 (3)	0.02782 (16)	
O4	0.28015 (13)	0.35571 (8)	0.49377 (4)	0.03210 (17)	
O5	0.83917 (16)	0.14540 (8)	0.43321 (4)	0.03596 (18)	
O6	0.65303 (16)	0.35172 (9)	0.39549 (4)	0.03701 (19)	
O7	1.15307 (17)	0.13761 (9)	0.33148 (4)	0.0417 (2)	
O8	1.06398 (15)	−0.08344 (8)	0.29288 (4)	0.03465 (18)	
H1A	1.257 (4)	−0.008 (2)	0.1562 (10)	0.074 (5)*	
H1B	1.548 (3)	−0.0609 (17)	0.1637 (8)	0.054 (4)*	
H1C	1.320 (3)	−0.1370 (19)	0.1966 (8)	0.054 (4)*	
H2A	0.179 (4)	0.122 (2)	0.6533 (10)	0.084 (6)*	
H3A	0.962 (4)	0.3630 (18)	0.5444 (8)	0.060 (5)*	
H4A	0.323 (4)	0.419 (2)	0.4681 (9)	0.070 (5)*	
H5	0.938 (4)	0.148 (2)	0.3926 (10)	0.072 (5)*	

### Atomic displacement parameters (Å^2^)

**Table:**

	*U*^11^	*U*^22^	*U*^33^	*U*^12^	*U*^13^	*U*^23^
C1	0.0228 (3)	0.0263 (4)	0.0218 (4)	−0.0020 (3)	0.0057 (3)	0.0023 (3)
C2	0.0223 (3)	0.0226 (4)	0.0233 (4)	−0.0009 (3)	0.0083 (3)	0.0002 (3)
C3	0.0237 (3)	0.0245 (4)	0.0228 (4)	−0.0023 (3)	0.0076 (3)	−0.0001 (3)
C4	0.0267 (3)	0.0266 (4)	0.0214 (4)	−0.0040 (3)	0.0066 (3)	−0.0039 (3)
C5	0.0221 (3)	0.0284 (4)	0.0215 (4)	0.0024 (3)	0.0093 (3)	0.0027 (3)
C6	0.0225 (3)	0.0333 (4)	0.0265 (4)	0.0000 (3)	0.0109 (3)	0.0026 (3)
N1	0.0387 (4)	0.0315 (4)	0.0269 (4)	0.0084 (3)	0.0162 (3)	0.0021 (3)
O1	0.0369 (3)	0.0426 (4)	0.0314 (4)	−0.0110 (3)	0.0154 (3)	−0.0113 (3)
O2	0.0484 (4)	0.0366 (4)	0.0335 (4)	−0.0202 (3)	0.0196 (3)	−0.0046 (3)
O3	0.0226 (3)	0.0297 (3)	0.0313 (3)	−0.0064 (2)	0.0107 (2)	−0.0030 (2)
O4	0.0259 (3)	0.0358 (4)	0.0347 (4)	0.0050 (2)	0.0120 (3)	0.0079 (3)
O5	0.0447 (4)	0.0324 (4)	0.0309 (4)	0.0075 (3)	0.0155 (3)	−0.0028 (3)
O6	0.0398 (4)	0.0399 (4)	0.0314 (4)	0.0031 (3)	0.0145 (3)	0.0089 (3)
O7	0.0490 (4)	0.0382 (4)	0.0380 (4)	−0.0039 (3)	0.0231 (3)	−0.0107 (3)
O8	0.0368 (3)	0.0314 (4)	0.0358 (4)	−0.0059 (3)	0.0189 (3)	0.0019 (3)

### Geometric parameters (Å, º)

**Table:**

C1—O1	1.2014 (11)	C5—O7	1.2499 (11)
C1—O2	1.3058 (10)	C5—C6	1.5153 (10)
C1—C2	1.5250 (10)	C6—N1	1.4746 (13)
C2—O3	1.4141 (10)	C6—H6A	0.9700
C2—C3	1.5364 (13)	C6—H6B	0.9700
C2—H2	0.9800	N1—H1A	0.953 (19)
C3—O4	1.4063 (11)	N1—H1B	0.909 (16)
C3—C4	1.5212 (10)	N1—H1C	0.914 (18)
C3—H3	0.9800	O2—H2A	0.93 (2)
C4—O6	1.2124 (11)	O3—H3A	0.870 (18)
C4—O5	1.3015 (11)	O4—H4A	0.81 (2)
C5—O8	1.2460 (11)	O5—H5	0.944 (19)
			
O1—C1—O2	125.71 (7)	O8—C5—C6	117.12 (7)
O1—C1—C2	124.10 (7)	O7—C5—C6	115.63 (8)
O2—C1—C2	110.19 (7)	N1—C6—C5	110.92 (7)
O3—C2—C1	108.12 (7)	N1—C6—H6A	109.5
O3—C2—C3	111.63 (7)	C5—C6—H6A	109.5
C1—C2—C3	109.07 (6)	N1—C6—H6B	109.5
O3—C2—H2	109.3	C5—C6—H6B	109.5
C1—C2—H2	109.3	H6A—C6—H6B	108.0
C3—C2—H2	109.3	C6—N1—H1A	109.2 (12)
O4—C3—C4	110.90 (7)	C6—N1—H1B	111.6 (10)
O4—C3—C2	110.33 (7)	H1A—N1—H1B	107.6 (15)
C4—C3—C2	110.42 (6)	C6—N1—H1C	115.4 (10)
O4—C3—H3	108.4	H1A—N1—H1C	105.0 (16)
C4—C3—H3	108.4	H1B—N1—H1C	107.6 (14)
C2—C3—H3	108.4	C1—O2—H2A	113.3 (13)
O6—C4—O5	126.76 (7)	C2—O3—H3A	108.3 (11)
O6—C4—C3	121.57 (8)	C3—O4—H4A	111.9 (14)
O5—C4—C3	111.66 (7)	C4—O5—H5	109.4 (11)
O8—C5—O7	127.24 (7)		
			
O1—C1—C2—O3	−1.72 (12)	C1—C2—C3—C4	177.86 (7)
O2—C1—C2—O3	177.86 (8)	O4—C3—C4—O6	4.99 (12)
O1—C1—C2—C3	−123.29 (10)	C2—C3—C4—O6	−117.63 (9)
O2—C1—C2—C3	56.29 (9)	O4—C3—C4—O5	−175.95 (8)
O3—C2—C3—O4	−64.51 (8)	C2—C3—C4—O5	61.43 (9)
C1—C2—C3—O4	54.91 (8)	O8—C5—C6—N1	23.03 (11)
O3—C2—C3—C4	58.45 (8)	O7—C5—C6—N1	−158.29 (9)

### Hydrogen-bond geometry (Å, º)

**Table:**

*D*—H···*A*	*D*—H	H···*A*	*D*···*A*	*D*—H···*A*
N1—H1*A*···O1^i^	0.953 (19)	2.20 (2)	2.9509 (11)	135.2 (16)
N1—H1*A*···O3^i^	0.953 (19)	2.21 (2)	2.9386 (11)	132.2 (15)
N1—H1*B*···O6^ii^	0.909 (16)	2.041 (16)	2.9188 (10)	162.0 (14)
N1—H1*C*···O7^ii^	0.914 (18)	2.172 (17)	2.9492 (13)	142.4 (14)
O2—H2*A*···O8^iii^	0.93 (2)	1.64 (2)	2.5473 (8)	167 (2)
O3—H3*A*···O4^iv^	0.870 (18)	1.849 (18)	2.7122 (8)	171.0 (16)
O4—H4*A*···O3^v^	0.81 (2)	2.09 (2)	2.7654 (10)	141.1 (18)
O4—H4*A*···O6	0.81 (2)	2.251 (18)	2.6743 (9)	112.9 (16)
O5—H5···O7	0.944 (19)	1.612 (19)	2.5459 (9)	169.2 (18)

Symmetry codes: (i) *x*+1/2, −*y*+1/2, *z*−1/2; (ii) −*x*+5/2, *y*−1/2, −*z*+1/2; (iii) −*x*+1, −*y*, −*z*+1; (iv) *x*+1, *y*, *z*; (v) −*x*+1, −*y*+1, −*z*+1.
